# Surgical Management of Inferior Turbinate Hypertrophy in the Era of Widespread Communicable Disease

**DOI:** 10.7759/cureus.34280

**Published:** 2023-01-27

**Authors:** Drew H Smith, Benjamin S Daines, Juliana Cazzaniga, Naveen D Bhandarkar

**Affiliations:** 1 Department of Otolaryngology - Head and Neck Surgery, Texas Tech University Health Sciences Center, Lubbock, USA; 2 Department of Otolaryngology - Head and Neck Surgery, Florida International University, Herbert Wertheim College of Medicine, Miami, USA; 3 Department of Otolaryngology - Head and Neck Surgery, University of California Irvine, Orange, USA

**Keywords:** nasal surgery, nasal obstruction, hypertrophy, turbinoplasty, aerosols, sars-cov-2

## Abstract

Inferior turbinate reduction procedures have been performed for decades. After significant evolution, turbinoplasty and other mucosal-sparing techniques have become the main method to successfully reduce turbinate hypertrophy. The debate of which technique produces the most effective and durable outcomes is ongoing. During this critical era of widespread communicable diseases, including but not limited to COVID-19, HIV, and hepatitis, additional attention is necessary to balance outcomes with a degree of generation of airborne particles when selecting a technique. This review article aims to identify the optimal method for inferior turbinate reduction that weighs both outcomes and aerosol production. The MEDLINE database was searched to discover relevant publications through August 2022. Key search terms included inferior turbinate hypertrophy, turbinate reduction surgery, turbinoplasty methods, surgical management of turbinate hypertrophy, surgical aerosol generation, COVID-19 surgery, surgery smoke plume, SARS-CoV-2 transmission during surgery, and nasal procedures COVID-19 aerosols. Surgical management of the inferior turbinates includes radiofrequency ablation (RFA), microdebrider-assisted turbinoplasty (MAIT), electrocautery, laser, and ultrasound. Piezo-assisted turbinoplasty and a turbinate-specific coblation wand are new additions to the literature. All techniques appear to improve patient symptoms of nasal obstruction. MAIT and RFA are comparable, although MAIT demonstrated better long-term outcomes in some studies and appears to generate fewer airborne particles. Studies evaluating the production of aerosols due to RFA are lacking. Ultrasound outcomes are also excellent and generate no aerosols, but the technique has not been compared against the microdebrider. Electrocautery can result in increased pain and crusting for patients and causes the highest amount of aerosols. Deficiencies of current studies, including a lack of comparison of aerosol generation, duration of follow-up, omission of outfracture, and inadequate randomized controlled trials among existing and new techniques, have limited the identification of the best inferior turbinate reduction method. Given the durability of MAIT and its minimal aerosol production, it can be reinforced as the most sensible technique until further evidence is available.

## Introduction and background

The inferior turbinates are important components of the respiratory and immune systems. Through a complex network of autonomic nerves that innervate vasculature and submucosal glands, they serve to provide appropriate warmth, humidification, and filtration of inspired air as well as necessary airflow resistance [1]. The inferior turbinate contributes nearly 16% of the heat and moisture needed to adequately condition inspired air for lung alveoli, the greatest contribution by any one structure in the airway [2]. In order to accomplish air-warming when the nose is exposed to cold temperatures, nasal airflow resistance increases as a result of augmented engorgement of blood vessels within the turbinates [1,3]. To fulfill the requirement for supplementary oxygen while exercising, an opposing action occurs: turbinate sizes decrease, resulting in decreased resistance by roughly 70 percent and an improved nasal cavity volume [4]. Healthy turbinates quietly play a significant role in our everyday activities.

Unfortunately, significant hypertrophy of the turbinates can occur, typically resulting in a primary symptom of nasal obstruction. While the worldwide prevalence of inferior turbinate hypertrophy is unknown, a large study of over 1900 patients with sinonasal complaints revealed that 72% suffered from hypertrophied turbinates, with 68% of that group reporting symptom scores that would categorize them as prime candidates for surgical intervention [5]. Surgery to address turbinate hypertrophy has existed for decades and involves removing all or part of turbinate tissue composed of bone, submucosa, and mucosa. In the 1970s, approximately 76% of surgical treatment was partial or total turbinectomy [6]. This practice was significantly reduced to roughly 14% during the 2000s due to complications of bleeding, significant crusting, and empty nose syndrome [7-9]. In the present day, techniques that focus on reducing submucosal tissue while maintaining mucosal function are more favored [9].

The extent of aerosol generation for each technique is particularly significant during this era of widespread communicable diseases, as the SARS-CoV-2 or COVID-19 virus and other viruses such as adenoviruses are known to spread via respiratory droplets that are prominent in nasal cavities [10,11]. Early reports have shown just how deadly this disease can be for practicing Otorhinolaryngologists with respect to the risk of infection or even death [12]. Standard surgical masks filter aerosolized particles greater than 5 µm and are unable to completely protect against all particles in surgical smoke which are less than 1.1 µm [13]. The aerosolized particles of SARS-CoV-2 are even smaller in size, ranging between 30-150 nm; accordingly, special consideration for safety must be given [13]. While the number of elective otolaryngology procedures performed during the early stages of the pandemic was significantly reduced, these elective caseloads have begun to recover toward pre-pandemic levels [14]. Furthermore, other airborne pathogens will continue to exist, posing a threat to surgeons and operating room staff. As such, techniques that limit aerosol generation are in the best interest of all.

The Centers for Disease Control and Prevention (CDC) recommends N95 or equivalent facemasks for aerosol-generating procedures, and hospitals may choose to use this level of personal protection equipment (PPE) regardless of a patient's COVID-19 status [15]. This may be limited by the availability of PPE for elective cases. With current testing lacking perfect accuracy in regard to sensitivity and specificity, this reinforces the support for minimal aerosol generation and optimal outcomes - choosing such a technique makes other factors, while still important to consider, less impactful.

Despite the significant amount of turbinate surgery performed worldwide each year, the single best technique has yet to be determined. Past randomized control trials (RCTs) comparing turbinate reduction techniques have been limited by quantity and quality. A 2010 Cochrane review was unable to identify any existing RCTs that met strict inclusion criteria for quality control [16]. More recent studies are now available for inclusion in analysis. Multiple recent studies have reviewed many of the modern techniques for inferior turbinate hypertrophy surgical intervention [17,18]. This paper will expand upon these previous reviews of existing literature in order to highlight known outcomes, new technique developments, current deficiencies in the literature, and most importantly, to evaluate which methods could reduce transmission of SARS-CoV-2 and other airborne vectors.

For this review article, the MEDLINE database was utilized to find and access relevant publications through August 2022. General search terms used include inferior turbinate hypertrophy, turbinate reduction surgery, turbinoplasty methods, surgical management of turbinate hypertrophy, surgical aerosol generation, COVID-19 surgery, surgery smoke plume, SARS-CoV-2 transmission during surgery, and nasal procedures COVID-19 aerosols. Turbinate reduction research articles and reviews that published outcomes, identified controversies, and highlighted deficiencies in the literature are included. Each new turbinate reduction method, as well as an update to approaches that were novel for previous reviews, is presented. All pertinent aerosol generation findings are discussed.

## Review

Early studies

One of the earliest comparisons of turbinate reduction methods was published in 2003 by Passali et al. [19]. This group evaluated the long-term efficacy of turbinectomy, laser cautery, electrocautery, cryotherapy, submucosal resection, and submucosal resection with lateral displacement (also known as outfracture). Results of the six-year study, one of the longest turbinate reduction follow-up periods to date, indicated that submucosal resection provided the greatest improvement in long-term nasal patency, as well as restoration of mucociliary clearance and normalized IgA production. The addition of lateral displacement to submucosal resection further enhanced long-term outcomes. Despite these conclusions, this study was excluded from the previously mentioned 2010 Cochrane review due to "failure to use stringent criteria for patient selection and allocation into groups" [16]. Regardless, many succeeding studies focused on submucosal resection techniques and will be examined. Aerosol generation in surgery has been investigated throughout the years, but recent events have inspired new publications [20-24].

Nasal endoscopic procedures, such as inferior turbinate reduction, may cause droplet formation via sneezing, vocalizing, or nasal spraying [25]. The risk of endonasal procedure droplet spread may be mitigated by the placement of an additional suction catheter [26]. In cadaveric studies, multiple rhinologic techniques, including the use of a microdebrider, high-speed drill, ultrasonic aspirator, and electrocautery, all caused significant increases in aerosols, especially of submicroparticles less than 1 µm [27]. It should be noted that suction devices and smoke evacuation systems were effective in reducing these aerosols. In living patients undergoing endonasal surgery, microdebrider and drill use were associated with significant increases in aerosolization, while cold instrumentation on suction was not [28]. This increase was only significant in the position of the operating surgeon and not for any other operating room positions.

Microdebrider-assisted inferior turbinoplasty (MAIT)

Following an incision to the anterior head of the inferior turbinate and the creation of a submucosal pocket, the microdebrider, composed of a rotating blade and suction, is inserted and then utilized to debulk excess soft tissue and/or bone [9,29]. This technique has been quite popular, given its ability to maintain mucosal function while minimizing complications [7].

The microdebrider has been shown to be of lower risk with respect to aerosol generation. Workman et al. demonstrated that use of a microdebrider for cadaveric endonasal procedures did not produce any detectable aerosols within the observed area immediately surrounding the nostrils [22]. In addition, a similar study evaluating use of the microdebrider on the posterior septum also did not yield any airborne aerosols [24]. Most importantly, no aerosolized droplets were discovered following a cadaveric septoplasty combined with MAIT [30]. However, other studies have called the lack of aerosolization by microdebrider into question. Sharma et al. and Murr et al. both demonstrated the generation of aerosols with microdebrider use during endonasal procedures [27,28].

Radiofrequency ablation (RFA)

RFA generally involves direct insertion of a coblator probe into the inferior turbinate and submucosal advancement for deliverance of thermal energy [29,31,32]. The alternating current produces high-frequency waves that agitate ions, resulting in shrinkage of the submucosal tissue through fibrosis [9,33,34]. The coblator probe or wand can be monopolar or bipolar, and multiple studies proved both to be equally effective and safe [35-37]. While comparative follow-up beyond two years is lacking, following proper surgical technique with either option appears practical.

Thermal energy from RFA acting on tissue produces a smoke plume, which is composed of tiny aerosols [21]. While no known trials have examined whether SARS-CoV-2 can be present in this plume, other viruses have been isolated in aerosol droplets within surgical smoke, including human papillomavirus (HPV), hepatitis B, and human immunodeficiency virus (HIV) [38-41]. Although the smoke in these studies was generated by means other than RFA, the theory that any coblator could produce an aerosolized virus is sensible but has not been directly assessed for turbinate reduction or other endonasal procedures. One study found that viable cancer cells were not present in RFA plumes following in vitro and in vivo experiments [42].

Electrocautery

Various approaches for electrocautery of the inferior turbinates exist in the literature. Some favor inserting a needle-tip or spatulated electrocautery probe immediately into the turbinate submucosa or following an opening incision [9,31,32,43,44]. Other reports describe the application of the electrode directly onto the turbinate surface [45,46]. Studies comparing submucosal versus mucosal techniques are not evident, though the importance of preserving the functional mucosa has been previously mentioned. Any electrocautery approach produces an electrical current that functions to heat a metal wire. The hot electrode can then be positioned on or in tissue, burning and destroying it [34]. The high voltage of electrocautery induces extremely high temperatures of up to 800°C; in contrast, the thermal energy generated during RFA generally does not exceed 90°C [47]. Given electrocautery's significant heat production, even the most skilled surgeon operating submucosally incurs a greater risk of damaging the overlying mucosa [47].

Similar to RFA, electrocautery also produces a substantial amount of surgical smoke known to contain fine aerosolized particles [20,48]. A recent study investigating electrocautery of the inferior turbinates also generated significant aerosols [24]. Suction was not used in this experiment to reduce aerosolized particles but could theoretically aid in practice. However, the same paper detailed airborne particle generation under distal suction conditions while drilling in the nasal cavity, and while suction reduced airborne particle concentration, it did not eliminate it [24]. Viral DNA from HIV and HPV have not been detected within electrocautery plume, even after exposure to culture medium [49,50]. It has been suggested that the electrocautery's high temperature may denature the viral DNA, effectively destroying it [49]. However, higher temperatures generated during electrocautery with higher wattage have been associated with greater airborne particulate during tonsillectomy [51]. Additional studies are needed to determine the risk of COVID-19 particulate spread during inferior turbinate electrocautery at different wattages.

Additional techniques

Alternate inferior turbinate reduction techniques that have been discussed in the literature include cryotherapy, laser, and ultrasound. Cryotherapy and laser surgery were particularly considered in the late 1990s and early 2000s. Cryotherapy involves applying a cryoprobe that induces a temperature between -12°C and -85°C to the turbinate, forming intracellular ice crystals that destroy cell membranes and induce tissue ischemia [7,9,52]. Laser surgery employs any of several different types of lasers, including carbon dioxide (CO2), neodymium:yttrium-aluminumgarnet (Nd:YAG), and holmium:yttrium-aluminumgarnet (Ho:YAG), among others, to excise excess turbinate tissue [53]. While laser surgery and cryotherapy have both been proven beneficial in addressing nasal obstruction due to hypertrophy, each targets the surface of the inferior turbinate and potentially disrupts its mucosa, limiting functionality [54,55]. Histopathologic analysis of inferior turbinate biopsies three months postoperatively revealed significant fibrosis, despite the reduction in inflammatory cells [56]. In addition to concerns over equipment costs and the need for repeated applications, patients also complained of significant crusting and pain [7,9]. Consequently, these techniques are no longer performed as frequently [9].

In 2010, a novel method utilizing ultrasound was reported to be feasible for turbinoplasty. Greywoode et al. applied an ultrasonic bone aspirator directly on the turbinate bone, emulsifying it and effectively reducing overall excess tissue without any complications [57]. Except for one comparative paper that also demonstrated ultrasound could successfully reduce nasal obstruction [44], additional follow-up studies are lacking.

While no known studies have examined aerosol generation of cryotherapy probes, viral airborne particles following the freezing process are unlikely. An ultrasonic bone aspirator is also safe, as a recent paper found no aerosol droplets were created during intranasal procedures while using this tool [30]. On the other hand, particles found in surgical smoke from laser use are even larger than those created by electrocautery [48,58]. Viruses, including HIV, HPV, and hepatitis B, have been aerosolized by lasers [39-41]. Laser smoke containing HPV can result in warts on those who are exposed to the plume, particularly in the nasopharynx [59]. Although these studies did not involve the inferior turbinates, it is reasonable to assume that the plume created by laser-turbinate surgery is potentially dangerous, and suction should be used to reduce particle concentration.

Emerging techniques

New turbinate reduction techniques have been presented. A 2019 paper by Robotti et al. detailed piezo-assisted turbinoplasty [60]. This technique uses piezoelectricity to make specific cuts in bone in order to remodel turbinate angulation, which can be preplanned via imaging. Bone is not removed and soft tissue is spared. It is important to note that this piezoelectric technique does not involve sonic waves, as some applicators do. In a sense, it is a more precise form of turbinate lateralization/outfracture. Additionally, a Bovie microtip was inserted through a small incision and applied to the turbinate submucosa for a supplementary reduction in what they termed "intramucosal microcauterization." This feasibility study of 157 patients with 12 months of follow-up resulted in improved breathing function, general maintenance of lateralization, and no complications. Longer follow-up and comparative studies are needed. A 2021 study by Ricciardiello et al. investigated quantic molecular resonance (QMR), utilizing low-frequency energy to disrupt bonds at a low temperature as a means of inferior turbinate reduction [61]. Despite functional improvements seen with QMR, the study lacked a control group or randomization, limiting the generalizability of results.

While not a novel technique, a new turbinate-specific coblator wand has recently been manufactured for RFA [62]. Compared to traditional RFA wands, this wand is slightly broader and has an active electrode at the tip, as well as suction and saline irrigation ports. The active electrode combined with the saline produces a unique plasma field within the tissue. This was designed to better control the amount of energy and heat delivered to the turbinate. However, no statistical difference has been found between typical RFA, RFA with the new coblator wand, and MAIT cohorts regarding patient outcomes, although larger and longer studies are needed [62,63].

There are no known evaluations for the aerosol-generating production of these recent techniques. Whether there is the creation of bone dust or other significant aerosolized particles with piezoelectricity is unknown. Although electrocautery, as mentioned earlier, involves aerosol generation, it is unknown whether the manner in which it is used in Robotti et al.'s study is safer than the traditional technique. As for the new coblator wand, the presence of the suction port could result in less aerosol emission during RFA, but it also requires further investigation.

Comparative studies

Studies comparing turbinate reduction techniques and their outcomes are numerous, with outcomes evaluated by several different measures. Common subjective measurements include the visual analog scale (VAS) and the Nasal Obstruction Symptom Evaluation (NOSE), both of which are patient questionnaires [64]. Objective methods include anterior rhinomanometry and acoustic rhinometry, among others, which calculate nasal airflow and patency, respectively, through specialized equipment [64]. However, patient-reported outcomes and objective measurement scores have been shown to have no correlation [65,66].

Prokopakis et al. compared CO2 laser, electrocautery, and RFA techniques among 2936 patients, the largest study to date [31]. Rhinomanometry and VAS scores for nasal obstruction, rhinorrhea, itching, and sneezing were measured at one month and one year post-surgery. All three cohorts had improved VAS scores at both follow-up times, although VAS scores were slightly worse at one year compared to one month. No statistical difference was found between the groups. While the authors describe a process of random selection for determining technique type, cohort size varied greatly: 1066 with CO2 laser, 664 for RFA, and 1206 with electrocautery. This study also mixed adult and pediatric patients, and patients whose surgery included outfracture were excluded.

In 2015, Shah et al. evaluated the outcomes of electrocautery and RFA [32]. This study was unique from many others in that surgeons performed both techniques on each of the 41 adult patients, electrocautery on the inferior turbinate in one nostril and RFA on the opposite side. Using VAS and acoustic rhinometry, researchers found that RFA resulted in significantly less pain and crusting in the initial post-operative weeks. Both procedures had improved subjective and objective nasal obstruction outcomes, but there was no statistical difference between them. Limitations of this study include a small sample size and only six weeks of follow-up, during which 40% of the patients were lost.

Gindros et al. examined the outcomes of ultrasound, RFA, and electrocautery [44]. This was the first study to compare ultrasound to other proven techniques. Sixty patients were evaluated for up to six months post-surgery via VAS, anterior rhinomanometry, and acoustic rhinometry. After six months, all groups showed subjective and objective improvement of nasal obstruction from pre-operative values, but ultrasound was statistically greater than RFA and electrocautery. RFA outcomes were slightly better than electrocautery but not enough to be statistically different. Additional ultrasound turbinoplasty studies, particularly with longer follow-up periods, are lacking.

A 2018 study by Harju et al. added a placebo procedure to their study in order to compare its outcomes with those of RFA, diode laser, and MAIT [67]. The placebo surgery consisted of taking 2-3 millimeter biopsies from the inferior turbinates and then repeatedly activating an RFA device near the patient, but without actually touching the patient. VAS scores appraising nasal obstruction, pain, crusting, and discharge were received from all 98 patients over three months. All cohorts reported statistically significant decreases of nasal obstruction severity, but RFA, diode laser, and MAIT provided an additional reduction compared to the placebo. Comparing the three actual procedures, none was statistically superior. A small cohort size, a short follow-up, and the lack of outfracture limit the study.

Mohamed et al. compared diode laser and electrocautery techniques for inferior turbinate reduction [68]. Both techniques achieved improvement in nasal obstruction, headache, and rhinorrhea postoperatively. Greater preservation of mucociliary function was achieved in the diode laser group, as evidenced by significantly improved saccharine testing. This demonstrates a potential role for diode lasers in inferior turbinate reduction. The study was limited by a small sample size of 42 patients and a lack of a control group except for saccharine testing.

RFA and MAIT have become increasingly more popular methods to address turbinate hypertrophy in the 2010s [69]. A landmark systematic review and meta-analysis by Acevedo et al. in 2015 compared outcomes of each technique [69]. Twenty-six total studies met the inclusion/exclusion criteria. The meta-analysis concluded that RFA and MAIT both demonstrate significant improvements in VAS and rhinomanometry-measured outcomes, but there is no significant difference between them for any outcome. This paper highlighted important deficiencies in the existing literature. Only 12 of the 26 studies included randomization, researcher blinding of performed technique occurred in solely three studies, and the median follow-up time was six months.

One RCT included in Acevedo et al.'s meta-analysis that has been given much attention is Liu et al.'s comparison of RFA and MAIT in 2009 [29]. While this paper (and all others) did not meet the inclusion criteria for the mentioned 2010 Cochrane review, those reviewers specifically noted that Liu's study came the closest to being includable. It was ultimately excluded for lack of randomization process details, which the reviewers were unable to obtain [16]. However, elements of Liu et al.'s methodology are superior to many other studies included in Acevedo et al.'s meta-analysis, and the results are significant. One hundred twenty patients were randomly assorted into equal size RFA and MAIT groups. Three years of follow-up were completed to evaluate outcomes by VAS (nasal obstruction, sneezing, rhinorrhea, and snoring), anterior rhinomanometry, and saccharin test for nasal mucociliary clearance. Only seven and 12 patients were lost to follow-up in the MAIT and RFA cohorts, respectively, at three years post-surgery. The MAIT cohort demonstrated significant improvement at six months, one, two, and three years after surgery for all outcome measures. RFA outcomes were only significant at six months and one year. They concluded that MAIT is more effective than RFA in the long term due to a more thorough removal of submucosal tissue. An additional study included in Acevedo et al.'s paper, while smaller and with only one year of follow-up, also found MAIT to have superior outcomes when compared with RFA [70]. An additional meta-analysis by Mirza et al. in 2020 comparing RFA and MAIT found better VAS scores in the MAIT cohort at three months, six months, one year, and two years postoperatively [71]. The MAIT cohort also had better anterior rhinomanometry scores at one and two years postoperatively.

Recently, Kankaanpää et al. utilized the Glasgow Health Status Inventory to evaluate patient-reported quality of life in 98 individuals who had randomly received turbinate treatment with MAIT, RFA, diode laser, or a placebo procedure [72]. All techniques, including the placebo, resulted in a quality-of-life improvement, but only MAIT had a statistically significant improvement over the placebo procedure at three months. However, researchers concluded that the number of patients in the placebo group was small and a larger study population may have resulted in the other techniques also achieving statistical significance.

LeConte et al. compared aerosolization between five common surgical techniques in endonasal surgery on cadaveric models, including electrocautery, MAIT, powered drilling, RFA, and cryotherapy [73]. Electrocautery, powered drilling, RFA, and cryotherapy all generated aerosols above the background, with most aerosols less than 1 µm in size. Overall, MAIT and cryotherapy produced significantly fewer aerosols than electrocautery and powered drilling. This study investigated the novel placement of an aerosol evacuator in the contralateral nasopharynx, which absorbed over 99% of all aerosols. This study was limited due to exclusive performance on cadavers and a lack of determination of whether or not aerosolized particles could carry the COVID-19 virus.

Existing literature deficiencies and future directions

Safety for all healthcare team members is paramount; therefore, understanding how to minimize the spread of communicable diseases in the operating room is crucial. While many previously mentioned tools used for turbinate surgery have been tested for propensity to generate aerosols, some, RFA for example, have not been studied specifically in turbinate procedures. An optimal study of aerosol generation for any technique should evaluate both for the creation of viable airborne SARS-CoV-2 droplets, for example, during inferior turbinate surgery, and the calculation of particle concentration with and without the use of suction. With any given technique, the use of suction in proximity is sensible if either aerosol generation has been confirmed or is unknown.

With no single turbinoplasty technique currently acknowledged as superior, addressing existing deficiencies in the literature may lead to improvements in the available evidence. Securing patient follow-up can be challenging for any research group, and very few existing studies include more than one to two years of follow-up. Passali et al. followed patients for up to six years, but 76% were lost by year six [18]. However, sufficient long-term follow-up is especially important for comparative outcomes, as this information can be used up front in technique selection to minimize the chance of recurrent hypertrophy and further procedures. Hill et al. presented strategies to achieve successful long-term follow-up [74]. Proposed actions include maintaining consistent data management staff, planning for adequate funding to address rising costs of data collection over time, keeping language open-ended with respect to time on consent forms, asking participants to provide contact information and relevant consent of at least three people who will always know their location, maintaining contact and relationships with patients between assessments even if remotely, and increasing effort in locating and re-establishing contact with lost participants. In addition, social media can also be used to continue contact, update patients of new findings, and remind them of important deadlines through a group Facebook page or Twitter account [74]. The design and use of smartphone apps could also be utilized to provide participants with relevant notifications.

Currently, outfracture or lateralization of the inferior turbinate is commonly performed in conjunction with any turbinate reduction surgery [75]. Nevertheless, outfracture is rarely included in formal studies comparing turbinoplasty techniques. Acevedo et al.'s systematic review of RFA and MAIT covered 26 studies in their final analysis, but only two of these included outfracture as part of the procedure [69]. Karakurt et al. investigated the addition or absence of outfracture in patients undergoing RFA inferior turbinate ablation and found significant functional improvements for the outfracture group [76]. Given its regularity in surgical practice, outfracture could be better evaluated in future studies.

Other future directions include larger cohort sizes and comparison studies that include less extensively studied and newer techniques. Although previous studies have measured the effectiveness of turbinate reduction when combined with other intranasal procedures, [46,77] studies assessing turbinoplasty technique outcomes when performed simultaneously with extranasal surgeries, such as adenotonsillectomy for relief of obstructive sleep apnea, could be helpful. Patient quality of life has been shown to increase after surgical turbinate reduction [78], and quantifying an amount based on technique could also be investigated further. 

Ideally, a multicenter approach featuring technique cohorts of over 100 patients, each with a follow-up period of greater than five years, would be completed. Assessment of aerosol generation during each surgery could be added. A study of that scale would likely be a significant addition to current evidence.

## Conclusions

While there is consensus that turbinectomy should generally be avoided, currently, there is no conclusive evidence as to which turbinate reduction technique has the best subjective or objective outcomes. While all techniques have demonstrated the ability to reduce nasal obstruction post-surgery, it appears that MAIT and RFA are more favorable, with the possibility that MAIT provides more enduring results. Other techniques, such as ultrasound and piezoelectricity, have shown advantageous outcomes in limited studies, but further investigation is needed. During this time of prevalent COVID-19 and with future endemic events likely, it is vital for the otolaryngologist to take aerosol generation into account during elective procedures. When considering the best available evidence on outcomes and potential for aerosol generation with each technique, MAIT appears to offer the most optimal balance of favorable outcomes with the lowest risk to the surgeon and operating room personnel. On the other hand, electrocautery and laser-assisted turbinate reduction may be better avoided due to the potential for direct damage to the mucosa and the ability to generate aerosol with potentially viable viral particles.
